# Correction: Estimation of the incubation period of COVID-19 in Vietnam

**DOI:** 10.1371/journal.pone.0248539

**Published:** 2021-03-09

**Authors:** The PLOS ONE Staff

The first author, Long V. Bui, is incorrectly noted as the corresponding author. The correct corresponding author is My Hanh Bui. My Hanh Bui’s email address is correctly listed: buimyhanh@hmu.edu.vn. The publisher apologizes for the error.
