# Supplementary material for: Estimation of the incubation period of COVID-19 in Vietnam
Source: PLoS One. 2020 Dec 23;15(12):e0243889. doi: 10.1371/journal.pone.0243889 (PMC7757798; doi:10.1371/journal.pone.0243889)
Supplement: Attachment — Submitted filename: Response to Reviewers.docx [file pone.0243889.s001.docx]

Dear Reviewer,

Thank you for your helpful comments. We have revised our paper accordingly and feel that your comments helped clarify and improve our paper. Please find our response (in blue) to reviewer’s specific comments (in black) below.

**Reviewer #1:**

1. The analyses were highly dependent on the epidemiological investigations for each case. Please describe the process of the data generation such as epidemiological investigations briefly to show the validity of the data such as the date of exposure and illness onset.

***Response:*** *Thank you for your comment. I have added details on the data generation: According to the report flow of the Ministry of Health and National Steering Committee for COVID-19 response, information of all laboratory-confirmed COVID-19 cases in Vietnam, which include patient number, travelling history, contact tracing and clusters, date of laboratory-confirmed, date of assertion, date and status of discharge was collected by the local centers for disease control and hospitals where patients were being admitted. The information then was reported daily to the Ministry of Health and officially made publicly available on the website httpp://ncov.moh.gov.vn.*

*This method was also used by several articles on COVID-19 in Vietnam, which were cited in our manuscript.*

2. The author compared the incubation period in other studies using the data from China. Can you please add the other studies using the data from out of china? For example, Korea (Lee H et al. Journal of Infection and Chemotherapy 2020), France (Slje H, hal-pasteur.achieve 2020), etc).

***Reponse:*** *Thank you very much. We have added relevant citation as you suggested. We did not cite Slje H, hal-pasteur.achieve 2020 because the article did not estimate the incubation period of COVID-19 in France, instead, the author used the incubation period of 5 days in a deterministic compartmental model stratified by age to describe the transmission of SARSCoV-2 in the French population.*

3. Please provide more detail of the limitation of this study, particularly on the small sample size to analyse.

***Response:*** *Thank you very much. We have added “In addition, the study could not analyze the differences in incubation period between age groups due to small sample size”.*

**Reviewer #2:**

1. How representative is the subsample? Data on age range and sex of the subsample was mentioned in the article, but not for the parent sample of 102 patients. Were their age/sex data comparable?

*Response: Thank you very much for your comment. We have added some lines that compare the age/sex of subgroup to the parent sample. “There was no statistical difference in mean age and sex distribution between sub-group for analysis and the entire group (p<0.05).”*

2. The authors attributed the large variation in incubation periods in their study to small sample size, but can these be contributed also by variations in exposure period and uncertainties in the date of infection (Figure 2)?

***Response:*** *Thank you very much. We’ve added this issue in the limitations: “The large variation in incubation periods in our study can also be attributed by variations in exposure period and uncertainties in the date of infection”.*

3. The authors commented that “Only one study by Leung [13] estimated longer incubation periods than our estimation”. But there are three other studies that gave estimates of longer mean incubation periods of COVID-19 in the order of 8 days: Kong T-K. Longer incubation period of coronavirus disease 2019 (COVID-19) in older adults. Aging Medicine 2020;3:102-109.

Qin J, You C, Lin Q, Hu T, Yu S, Zhou X-H. Estimation of incubation period distribution of COVID-19 using disease onset forward time: a novel cross-sectional and forward follow-up study. Sci Adv. 2020. <https://advances.sciencemag.org/content/early/2020/08/07/sciadv.abc1202>

***Response:*** *Thank you very much. We have added relevant citation as you suggested. We did not cite (Slje H, hal-pasteur.achieve 2020) because the article did not estimate the incubation period of COVID-19 in France, instead, the author used the incubation period of 5 days in a deterministic compartmental model stratified by age to describe the transmission of SARSCoV-2 in the French population.*

4. The authors recommended the quarantine period should be extended up to three weeks based on their study finding of 97.5th percentile 13.0, CI 10.6–20.7. But would they also comment on the wide CI of 10.6-20.7 and the reason for the uncertainty?

***Response:*** *Thank you very much. We’ve added one reference, suggesting that the incubation period of CVOID-19 may be up to 24 days “Prior studies also suggested that the incubation period in some cases can be up to 24 days [24]”.*

Thank you again for your constructive manuscript, that absolutely improve our manuscript.

We hope reviewers take our revised version into your consideration.

We look forward to hearing positive feedback from you.

Sincerely Yours

Bui My Hanh
