# Supplementary material for: Estimation of the incubation period of COVID-19 in Vietnam
Source: PLoS One. 2020 Dec 23;15(12):e0243889. doi: 10.1371/journal.pone.0243889 (PMC7757798; doi:10.1371/journal.pone.0243889)
Supplement: Attachment — Submitted filename: Response to Reviewers.docx [file pone.0243889.s002.docx]

Dear Reviewers,

Thank you for your helpful comments. We have revised our paper accordingly and feel that your comments helped clarify and improve our paper. Please find our response (in blue) to reviewer’s specific comments (in black) below.

**Reviewer #1:**

The authors reflect all of the comments in the revised manuscript; The readibility is improved and looks better.

***Response:*** *Thank you for your comment.*

Reviewer #2:

My review comments have been addressed and the manuscript is acceptable for publication after correction of one error and a typo:

Line 157 error: Lee et al [10] should not be quoted as a study showing longer incubation period than the authors' study finding, because the median incubation period of Lee et al [10] is shorter at 3 days. It can nevertheless be quoted under Line 155 together with other studies showing shorter incubation period compared with the authors' finding.

Line 112 typo: "of those" is duplicated.

***Response:*** *Thank you for your comment. We have deleted the duplicated “of those”. We have moved the reference of Lee et al. to studies estimating shorter incubation time than ours and revised the range of shorter estimation from 3 – 5.6 days.*

*We have made overall editing to the manuscript to make sure all typos and wordings are correct.*

Thank you again for your constructive manuscript, that absolutely improve our manuscript.

We hope reviewers take our revised version into your consideration.

We look forward to hearing positive feedback from you.

Sincerely Yours

Bui My Hanh
